# Agathisflavone as a Single Therapy or in Association With Mesenchymal Stem Cells Improves Tissue Repair in a Spinal Cord Injury Model in Rats

**DOI:** 10.3389/fphar.2022.858190

**Published:** 2022-04-05

**Authors:** Ravena P. do Nascimento, Lívia B. de Jesus, Markley S. Oliveira-Junior, Aurea M. Almeida, Eduardo L. T. Moreira, Bruno D. Paredes, Jorge M. David, Bruno S. F. Souza, Maria de Fátima D. Costa, Arthur M. Butt, Victor Diogenes A. Silva, Silvia L. Costa

**Affiliations:** ^1^ Laboratory of Neurochemistry of Cellular Biology, Department of Biochemistry and Biophysics, Institute of Health Sciences, Federal University of Bahia, Salvador, Brazil; ^2^ Department of Anatomy, Pathology and Veterinary Clinics, Hospital of Veterinary Medicine, Federal University of Bahia, Salvador, Brazil; ^3^ Center for Biotechnology and Cell Therapy, São Rafael Hospital, D’Or Institute for Research and Education, Salvador, Brazil; ^4^ Department of General and Inorganic Chemistry, Institute of Chemistry, Federal University of Bahia, Salvador, Brazil; ^5^ Gonçalo Moniz Institute, FIOCRUZ-BA, Salvador, Brazil; ^6^ INCT–Translational Neuroscience (INCT-TN, BR), Salvador, Brazil; ^7^ School of Pharmacy and Biomedical Sciences, University of Portsmouth, Portsmouth, United Kingdom; ^8^ INCT for Excitotoxicity and Neuroprotection (INCT-EN, BR), Salvador, Brazil

**Keywords:** MSCs, mesenchymal stem cells, agathisflavone, acute spinal cord injury, neurotrophins, regeneration

## Abstract

Agathisflavone is a flavonoid with anti-neuroinflammatory and myelinogenic properties, being also capable to induce neurogenesis. This study evaluated the therapeutic effects of agathisflavone—both as a pharmacological therapy administered *in vivo* and as an *in vitro* pre-treatment aiming to enhance rat mesenchymal stem cells (r)MSCs properties–in a rat model of acute spinal cord injury (SCI). Adult male Wistar rats (*n* = 6/group) underwent acute SCI with an F-2 Fogarty catheter and after 4 h were treated daily with agathisflavone (10 mg/kg ip, for 7 days), or administered with a single i.v. dose of 1 × 10^6^ rMSCs either unstimulated cells (control) or pretreated with agathisflavone (1 µM, every 2 days, for 21 days *in vitro*). Control rats (*n* = 6/group) were treated with a single dose methylprednisolone (MP, 60 mg/kg ip). BBB scale was used to evaluate the motor functions of the animals; after 7 days of treatment, the SCI area was analyzed after H&E staining, and RT-qPCR was performed to analyze the expression of neurotrophins and arginase. Treatment with agathisflavone alone or with of 21-day agathisflavone–treated rMSCs was able to protect the injured spinal cord tissue, being associated with increased expression of NGF, GDNF and arginase, and reduced macrophage infiltrate. In addition, treatment of animals with agathisflavone alone was able to protect injured spinal cord tissue and to increase expression of neurotrophins, modulating the inflammatory response. These results support a pro-regenerative effect of agathisflavone that holds developmental potential for clinical applications in the future.

## Introduction

Spinal cord injury (SCI) is a devastating neurological condition, with a global incidence of 10.4–83 cases/million/year (Karsy and Hawryluk, 2019). Treatment of SCI requires multidisciplinary action in the acute phase and also for secondary complications associated with long-term injury (Liu et al., 2017). Currently, routine therapy employed in the early stages of SCI mainly involves surgical procedures combined with high doses of methylprednisolone (MP) for the inhibition of lipid peroxidation and maintenance of the blood barrier of the spinal cord, although this treatment is controversial (Cabrera-Aldana et al., 2017; Liu et al., 2018). Mesenchymal stem cells/stromal cells (MSCs) have arisen as a treatment for SCI in animal models and in patients, either alone or in association with drugs (Mendonça et al., 2014; Assinck et al., 2017; Larocca et al., 2017; Allahdadi et al., 2019; Jin et al., 2019). Recent studies have shown that MSCs release exosomes that attenuate apoptosis and inflammation; they also suppress glial scarring, attenuate lesion size and promote axonal regeneration culminating in better behavioral recovery (Yin et al., 2019; Yuan et al., 2019). However, direct transplantation of MSCs to target tissues remains challenging, as low cell survival rates, cell dedifferentiation, immune rejection and tumor formation can all compromise the efficacy of this therapy (Caplan et al., 2019).

Flavonoids stand out as an important class of natural antioxidants with demonstrated neuroprotective effects in animal models of SCI, including curcumin (Akar et al., 2017), huangqin (Zhang et al., 2018) and baicalin (Kang et al., 2018). In addition, the flavonoid apigenin has been reported to significantly reduce side effects in an animal model of SCI (Zhang and Liao, 2014). Agathisflavone, a biflavonoid composed of two apigenins, exhibits anti-inflammatory (dos Santos Souza et al., 2018), neurogenic and neurodifferentiating activities *in vitro*, as well as a neurogenic effect on embryonic stem cells and multipotent stem cells (Paulsen et al., 2011). We therefore postulated that agathisflavone may be a useful adjuvant to MSC therapy in SCI. Our results show that the infusion of rat (r)MSC treated with agathisflavone increased the production of neurotrophic and anti-inflammatory factors in a rat model of SCI.

## Materials and Methods

**FIGURE 1:**
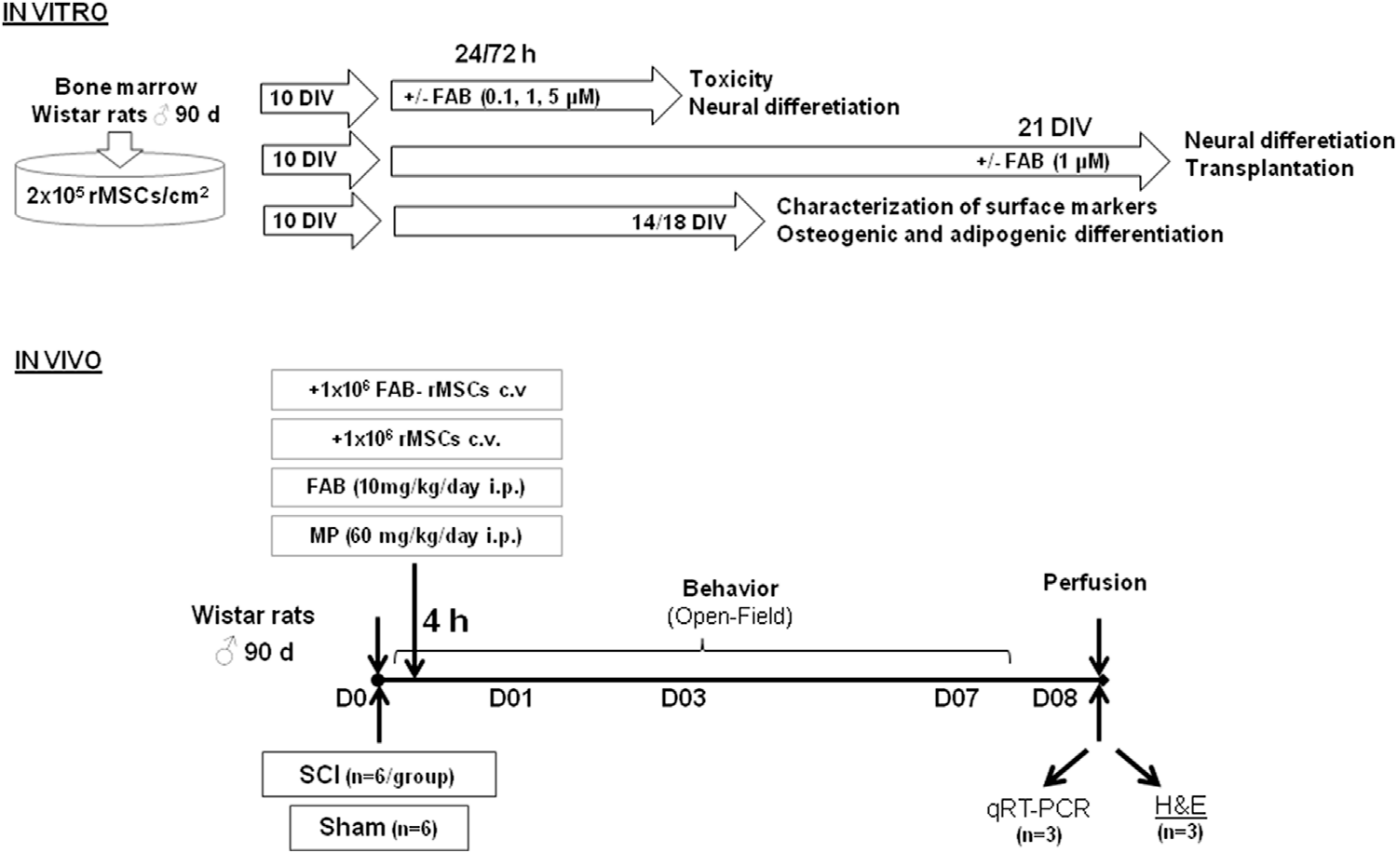
Experimental design showing model adopted to investigate effects of rat bone marrow mesenchymal stem cells (rMSCs) and agathisflavone (FAB) treatments in a model of spinal cord injury (SCI). Days *in vitro* (DIV).

A summary of the experimental design is presented in Figure 1.

### Animals

Thirty-six 30-day-old male Wistar rats were obtained from the animal facility of the Center for Biotechnology and Cell Therapy, São Raphael Hospital and kept in the vivarium of the Institute of Health Sciences of the Federal University of Bahia, Salvador, Brazil. They were maintained in a controlled environment (12 h light/dark cycle, 22 ± 1°C, with access to food and water *ad libitum*). Rats were used experimentally after reaching 3 months of age and weight ranging from 250 to 280 g. All animal procedures were performed according to the National Institute of Health (NIH) Guide to the Care and Use of Laboratory Animals and approved by the Animal Use Ethics Committee of the Institute of Health Sciences of the Federal University of Bahia (process number 117/2017).

### Purification and Culture of rMSC

Rat mesenchymal stem cells were isolated using the method originally described by Friedenstein et al. (1974), which is based on their ability to adhere to plastic. Briefly, bone marrow aspirate was made from isolated femur bones of male adult rats and then processed using the hydrophilic polysaccharide Ficoll^®^ as the centrifugation gradient. For this, bone femur epiphysis was minimally cut and then a needle was attached to a syringe containing DMEM medium and inserted into the epiphysis to wash the bone cavity. This bone marrow was then collected, resuspended in DMEM medium in a 2-ml volume added to a 10 ml tube falcon and centrifuged for 10 min at 1,750 centrifugal g force (Hettiche^®^ Universal 320R Centrifuge). Then, the pellet was resuspended in a volume of 2 ml of DMEM medium and transferred to a 10-ml tube falcon. 1 ml of the Ficoll^®^ density gradient was added and then centrifugation was performed for 15 min at 4,820 centrifugal g force (Hettiche^®^ Universal 320R Centrifuge). After that, the ring of cells at the Ficoll^®^/DMEM interface was collected and resuspended in DMEM medium in volume 1: 5 ml cells: medium. Cells were collected and plated in Dulbecco’s Modified Eagle Medium (DMEM), enriched with fetal bovine serum (FBS) and antibiotics (100 IU/Ml penicillin G, 100 µg ml^−1^ streptomycin, Gibco). After 24 h, the culture medium was changed to remove non-adherent cells. The remaining cells were grown in a humid incubator (37°C, 5% CO_2_) and the culture medium was changed every 2–3 days until cells reached a confluence of about 80%, which occurred around 10 days *in vitro* (DIV). Cells were then trypsinized and expanded by subsequent passages. In this work, all the tests were run with passage 3 rMSCs and in triplicates (Figure 1).

### Characterization of rMSC

Rat mesenchymal stem cells (rMSC) were characterized according to the criteria of the International Society for Cellular Therapy (Horwitz et al., 2005; Caplan 2009), namely expression of specific markers (CD29, CD73, CD90, CD11b, CD34 and MHC-II), measured by flow cytometry (Complementary Material I), and their adipogenic and osteogenic potential in culture (Complementary Material II). In brief, 1 × 10^6^ rMSC were cultured for 10 DIV in 10-mm diameter plates (TPP^®^) in DMEM with 10% FBS, antibiotics (100IU/Ml penicillin G, 100 µg/ml^−1^ streptomycin, Gibco^®^), 10 M dexamethasone (Sigma^®^). For adipogenic differentiation, the medium was supplemented with insulin (2.5 μg/ml), indomethacin (100 μM) and 3-isobutyl methylxanthine (0.5 mM); as for osteogenic differentiation, the medium was supplemented with ascorbic acid (50 μg/ml) and β-glycerolphosphate (3.15 mg/ml). After 11 days, cells were fixed with 4% paraformaldehyde for 30 min and stained for 5 min at room temperature with Oil Red O for adipocytes, or alizarin red for osteoblasts (Muniswami et al., 2018). The cultures were then washed with PBS and examined under bright field microscopy (Figure 1).

### Flavonoid Treatment of rMSC

Agathisflavone was extracted from the aqueous extract of the leaves of *P. pyramidalis* Tull as previously described (Mendes et al., 2000; Bahia et al., 2005; Bahia et al., 2010). Agathisflavone was dissolved in dimethyl sulfoxide (DMSO; Sigma^®^) to yield a 100-mM stock solution that was stored and protected from light at −4°C. As described above, rMSC were cultured to passage (P)3 and plated at a density of 2 × 10^5^ cells/cm^2^ and cultured for 24 h, prior to treatment with agathisflavone diluted directly into the culture medium to achieve the final concentration (see below). Multiple analyses were performed after 1–21 days in culture (see below).

### Analysis of rMSC Viability

Viability of rMSC was assessed using the MTT assay (3-(4,5-dimethylthiazol-2-yl)-2,5-diphenyltetrazolium bromide test; Sigma^®^, St. Louis, MO), which is based on the conversion of the yellow MTT by the dehydrogenases of living cells to purple-colored formazan (Hansen et al., 1989). The experiment was performed in 96-well plates (Kasvi^®^, Brazil) of cultured rMSC. Cells were incubated in 0.01% DMSO (control) or with agathisflavone (0.1, 1.0 or 5 µM). 24 h or 72 h after treatment, cells were incubated with MTT (1 mg/ml) for 2 h. Subsequently, cells were lysed with 20% (vw^−1^) with sodium dodecyl sulfate (SDS) and 50% (vv^−1^) dimethylformamide (DMF) (pH 4.7). The plates were incubated overnight at 37°C to dissolve the formazan crystals and the optical density of each sample was measured at 590 nm using a spectrophotometer (Thermo Plate-Reader^®^). At least three independent experiments were performed with eight replicate wells for each analysis. MTT results are expressed as percentages of cells converting MTT compared to control cultures (considered as 100%).

### Characterization of rMSC Morphology

Based on the results of the MTT test, morphological analysis was performed in cultures treated with agathisflavone at 0.1 and 1 µM for 72 h, compared to DMSO controls. 72 h after treatment, cultures were washed three times with phosphate buffered saline (PBS), fixed and permeabilized with cold methanol at −20°C for 20 min and analyzed by Rosenfeld staining, by adding ed to the plates previously fixed with cold methanol at –20°C in enough volume to completely cover the cells. After 3 min, 20 drops of distilled water were added to the dye solution and the stain was allowed to act for a further 20 min at room temperature. The plates were then washed with distilled water, dried and analyzed by means of light microscopy.

### Characterization of Neural and Glial Markers in rMSC

In order to investigate whether the flavonoid agathisflavone induces phenotypic changes in rMSC associated with neural and glial differentiation, immunocytochemistry was performed 72 h after treatment with 1 µM agathisflavone, compared to DMSO-treated controls. Immunocytochemistry was performed as described by dos Santos Souza et al., 2018. Briefly, cells were fixed with 4% paraformaldehyde (PFA) and 4% sucrose for 20 min and permeabilized with 0.2% 4- (1,1,3,3-tetramethylbutyl) phenyl polyethylene glycol (Triton X-100, Merck^®^) for 5 min at room temperature. After permeabilization, cells were blocked with 5% bovine serum albumin (BSA; Invitrogen^®^) in PBS (blocking solution) for 1 h and incubated overnight at room temperature with primary antibodies diluted in blocking solution: mouse anti-β-III-tubulin antibody, 1:1,000 (Promega^®^, Madison, WI); anti-rabbit glial fibrillary acidic protein (GFAP) antibody, 1:200 (Dako Corporation, Glostrup^®^, Denmark). After incubation with primary antibodies, cells were washed extensively with PBS and incubated with secondary antibodies for 1 h at room temperature. Secondary antibodies were purchased from Molecular Probes^®^ (Eugene, OR): Alexa fluor 488 conjugated goat anti-mouse IgG (1: 400), Alexa fluor 546 conjugated goat anti-rabbit IgG (1: 1,000). Negative controls were performed by omitting the primary antibody during immunostaining and, in all cases, no reactivity was observed when the primary antibody was absent. Cell preparations were mounted directly on 3,4,5-trihydroxy benzoate (N-propyl gallate, Sigma-Aldrich^®^) and visualized using a Leica^®^ EBQ 100 fluorescence light microscope.

### SCI Experimental Design

Male Wistar rats were divided into six groups (*n* = 6 animals/group): sham; spinal cord injury (SCI); treated with a single application (via caudal vein) of 1 × 10^6^ 21 DIV control rMSCs; treated with a single application (via caudal vein) of 1 × 10^6^ 21 DIV agathisflavone-treated rMSC; treated with one single dose of methylprednisolone (MP, 60 mg/kg i. p.); or treated daily with agathisflavone (10 mg/kg i.p.) once a day, at the same time for 7 days. Caudal vein administration was performed based on studies with other flavonoids in an SCI model (Zhang et al., 2014; Akar et al., 2017).

On the day of surgery, the animals were individually weighed to obtain the volume of the anesthetic mixture. The combination used was ketamine chloridate (ketalar^®^) 75 mg/kg IP and xylazine chloridate (rompum^®^) 10 mg/kg IP. Spinal cord injury was induced as previously described (Vanický et al., 2001). We performed trichotomy and asepsis of the skin in the thoracic region with Betadine^®^. Then, a midline incision of 20 mm was made in the thoracic region, and the spine was exposed. Laminectomy was performed at vertebral level T-10, exposing the dorsal cord. A Fogarty F-2^®^ catheter was inserted into the dorsal epidural space through a small hole in the T10 spinal arch, cranially advanced at the level of the T8-9 spine and inflated for 5 min with the aid of a Hamilton^®^ syringe and 15 µl of saline solution (Figure 1). After surgery, animals were submitted post-operative care: they were kept in individualized cages heated to a constant temperature of 27°C to avoid hypothermia, received saline solution subcutaneously (3 ml) and were monitored at least 4 times a day to assess the presence of pain in the post-procedure and for the emptying of the urinary bladder. Until normalization of excretory functions, bladder massage was performed 4 times a day to assist urinary bladder emptying. All animals received topical application of rifocina^®^ to combat topical infections (Rifamycin sodium salt, 10 mg/ml, spray). During the experimental period, the animals were monitored for feeding, water intake and excretory function (urine and feces). They all received water and food *ad libitum.*

### Motor and Weight Variation Analysis of Animals Submitted to Acute Spinal Cord Injury

Animals were examined and weighed do determine their general health on days 0, 1 and 7, where day 0 refers to the day of surgery. Motor assessment was performed using the Basso, Beattie, Bresnahan scale (BBB) (Basso et al., 1995), by observing the movements of the hip, knee, ankle joint, trunk position, tail and hind legs. From these observations, points were assigned from zero to 21, with zero corresponding to the total absence of movements and 21 to the presence of normal movements (Barros Filho and Molina, 2008). The animals were placed in an open field, observed and filmed for 10 min. Motor assessment was performed on days 0, 1 and 7 post-surgery.

### Histopathological Analysis of the Spinal Cord by Hematoxylin and Eosin

For morphological analysis, rats were intracardially perfused with saline solution and fixed with 4% PFA for 10 min under terminal anesthesia. A ketamine/xylazine mixture (up to 75 mg/kg body weight ketamine and 10 mg/kg xylazine body weight) was administered by intraperitoneal injection (27-gauge needle and a cc syringe). Additional anesthetic administration was performed as needed during each operation to maintain an anesthesia surgical plan. Once the animal reached a surgical plan for anesthesia, surgery and perfusion were performed. Spinal cords were removed and fixed in 10% formalin buffer for approximately 7 days at RT. After fixation, the spinal cord tissue was dehydrated and included in paraffin. It was then halved in the posterior median sulcus and embedded in paraffin. Serial longitudinal 4-µm sections were cut on a microtome at the level of the SCI, between 5 mm before the T7 vertebra and 5 mm after the T8 vertebra. Serial sections were dewaxed in ascending xylene stained using hematoxylin and eosin (H&E) and mounted on slides. The slides were evaluated under light microscope by a pathologist blinded to the groups. The histopathological evaluation was evaluated by the semiquantitative classification system, as described by Ustun et al. (2014) Table 4. Quantification of macrophages was made in ten randomly assigned photomicrographs (50-μm range) for each experimental group.

### Molecular Analyses of Neuroinflammatory Profile in the Site of the SCI

Three spinal cord samples were randomly assigned to each group of animals for ribonucleic acid (RNA) extraction using Trizol^®^ reagent (Invitrogen, Life Technologies™) according to the manufacturer’s specifications. cDNA synthesis was performed using SuperScript^®^ VILO ™ Master Mix (Invitrogen, Life Technologies ™) following the manufacturer’s instructions. Quantitative real-time polymerase chain reaction (RT-qPCR) was performed using Taqman^®^ gene expression assays (Applied Biosystems, CA, United States) containing two primers to amplify the sequence of interest and the Taqman^®^ MGB-specific probe labeled FAM fluorophore with TaqMan^®^ Universal Master Mix II (Invitrogen, Life Technologies ™). Assay identifications for the genes quantified in this study were: Arginase 1 (RN00681090_m1), nerve growth factor/NGF (RN01533872_m1) and glia-derived neurotrophic factor/GDNF (RN00569519_m1), both distributed by Thermo Fisher (TaqMan^®^ Gene Expression assay). RT-qPCR was performed using the QuantStudio ™ 7 Flex Real-Time PCR System instrument (Applied Biosystems, CA, United States). Thermocycling conditions were performed according to the manufacturer’s specifications. Β-actin (Mm00607939_S1) and HPRT1 (Mm01545399_m1) were used as reference genes (endogenous controls) for normalization of gene expression data. The analysis of real-time polymerase chain reaction data was based on Schmittgen and Livak (2008), using the 2-ΔΔCt method. All tests were performed in triplicate.

### Statistical Analysis

Statistical analyses were performed with the GraphPad Prism software version 5.00 for Windows. Data were shown as means with standard deviation or means with range according to their distribution, analyzed with the Shapiro–Wilk normality test and Skewness (normal: < 1 or > −1) and Kurtosis (normal: < 2 or > −2) calculation. In addition, according to the distribution, parametric or nonparametric statistic tests were chosen. The most appropriate test for each experiment was used, and this information is given in the respective result. All analyses were carried out with triplicates.

## Results

### Characterization of Rat Mesenchymal Stem Cells (rMSC)

First, we characterized rMSCs isolated from adult rat femur bone marrow on the third passage (P3), by their differentiation and surface expression of key markers measured by flow cytometry (Supplementary Figures S1, S2). rMSCs adhered to plastic and presented a fibroblast-like morphology 7–10 days after being seeded onto culture flasks (Supplementary Figure S1A). rMSC submitted to adipogenic differentiation displayed lipid droplet formation after 9 days, and this was remained up to 15 days, as determined by Oil red O staining (Supplementary Figure 1SB). rMSC submitted to osteogenic differentiation displayed calcium deposition during the first 10 days after induction and remained for 21 days, characterizing matrix mineralization as determined by alizarine red staining (Supplementary Figure S1C). As controls for both differentiation protocols, cultures were cultivated with expansion medium during the entire protocol and, as expected, showed no formation of lipid vacuoles or calcium deposits; the doubling time of these cells was approximately 60 h and linear between P3 and P5. In addition, flow cytometry demonstrated that 68.5, 67.6 and 99.7% of the rMSC cell population were respectively positive for CD90 (VLA-β integrin), CD73 and CD29, and the vast majority (>95%) were negative for typical hematopoietic and endothelial cell markers CD11b (immune cell marker–integrin Mac-1) and CD34, with negligible expression of MHC-II, respectively (Supplementary Figure S2 and Table 1). Together, these findings demonstrate our protocols provided cultures of characteristic MSC in corroboration with other studies (Caplan, 2009; Dariolli et al., 2013).

**TABLE 1:** Characterization of mesenchymal stem cells by flow cytometry.

	% Positive cells	% Negative cells
CD11b+	0.8	99.2
CD34	3.33	96.67
MHC II	3.48	96.52
CD29	99.7	0.3
CD73	67.6	32.4
CD90	68.9	31.1

### Effects of Agathisflavone on Morphology and Viability of rMSC

**FIGURE 2:**
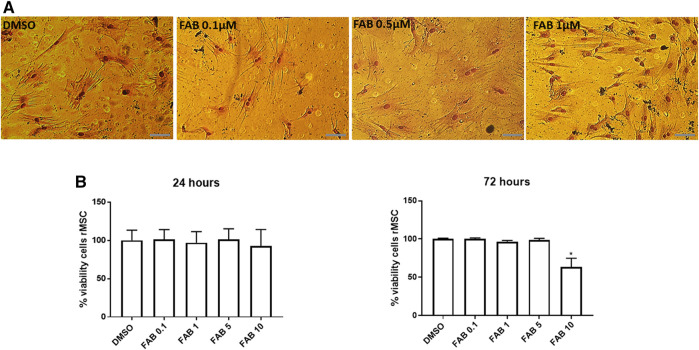
**(A)** Morphological aspect of 30 days *in vitro* (DIV) of rMSCs of cultures in control conditions (0.01% DMSO) and 72 h after treatment with FAB (0.1, 0.5 and 1 µM); Rosenfeld staining; obj. x40, scale bar 50 µm. After treatment with 0.1 and 0.5 µM FAB, the cells presented a flat polygonal morphology similar to that of the control (DMSO). However, after treatment with 1 µM FAB, the cells presented Y-shaped extensions. **(B)** Analysis of viable rMSCs of cultures in control conditions (0.01% DMSO) and 24 and 72 h after treatment with FAB (0.1, 1, 5 and 10 µM), showing no toxicity in 24 h and toxicity only for FAB 10 µM 72 h after treatment; MTT test; Each graph is representative of three independent experiments and the data are expressed as means ± standard deviation. An ANOVA one-way test followed by Turkey’s test for multiple comparisons was performed.

The next step was to evaluate the potential toxicity of agathisflavone on P3 rMSC. Culture of rMSC with 0.1–5 µM agathisflavone had no effect on cell viability after 24 h. A reduction on cell viability was observed only in 72 h for a treatment with agathisflavone 10 µM, as determined by the MTT assay, compared to control cultures treated with 0.01% DMSO vehicle (Figure 2B). The effects of agathisflavone on rMSC morphology was evaluated by Rosenfeld’s staining (Figure 2A). In control cultures treated with 0.01% DMSO vehicle, rMSC was presented as large cells with a flat polygonal morphology, with very short processes or without processes, and a clearly visible nucleus, typical of mature cells with the classification of “smaller stem potential” (Sekiya et al., 2002; Neuhuber et al., 2008). Cells treated with 0.1 or 0.5 µM agathisflavone for 72 h presented the same pavement morphology as control cultures, whereas following treatment with 1 µM agathisflavone a subpopulation of rMSC displayed a very clear cell body, refringent nucleus and long and thin Y-shaped extensions. It was also observed that some cells presented an astrocyte-like polyhedral morphology, with processes extending from their cell body. The cells retain their characteristic morphology, but at 1 µM agathisflavone evidently altered rMSC morphology, which may be indicative of inducing cellular pluripotency.

The MTT results demonstrate that agathisflavone is non-toxic for rMSC at any of the concentration used (0.1, 1 or 0.5 µM agathisflavone) after 24 h.

### Effects of Agathisflavone on rMSC Differentiation

**FIGURE 3:**
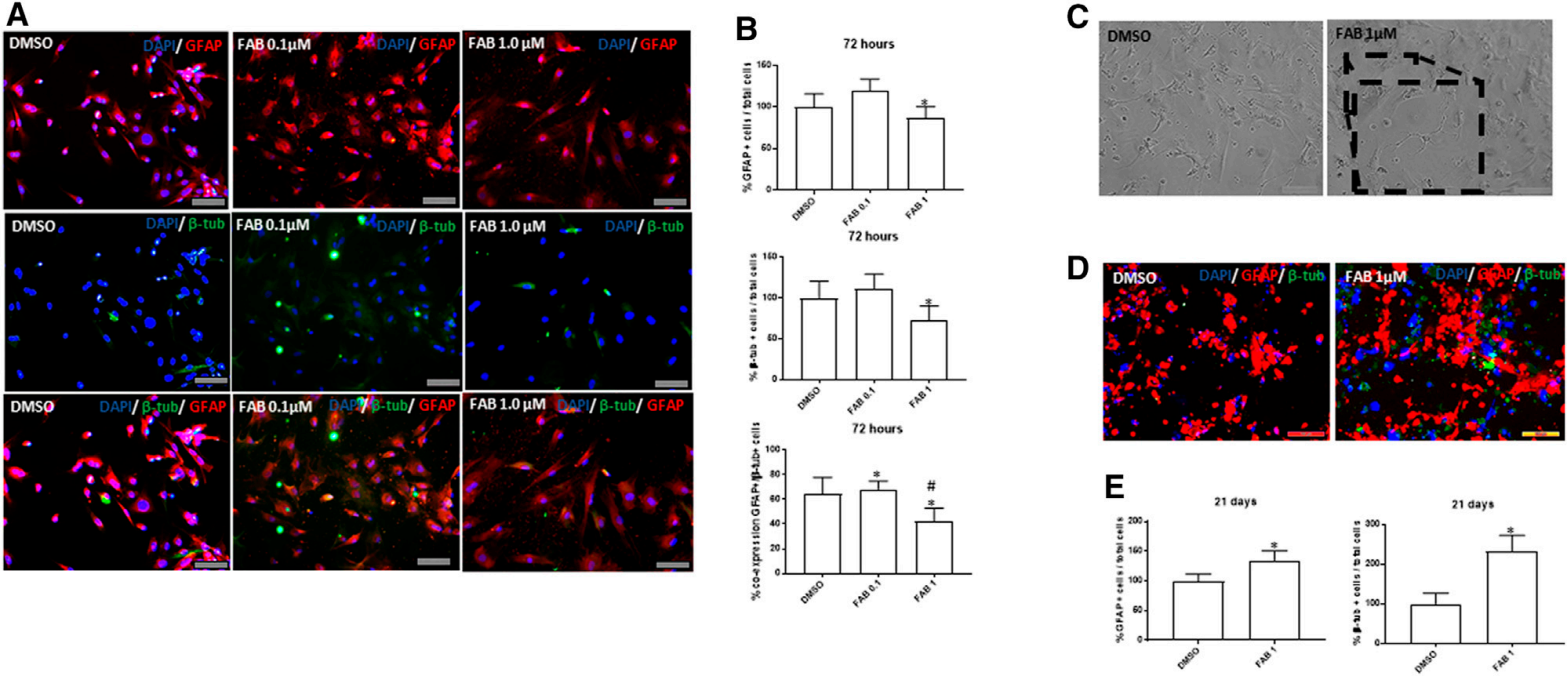
Analysis of the expression of neural markers GFAP (red) and β-tubulin III (β-tub, green) in rat bone marrow-derived mesenchymal stem cells (rMSCs). **(A)** Photomicrographies of cultures in control conditions (0.01% DMSO) and 72 h after treatment with agathisflavone (0.1, 1 µM FAB); immunocytochemistry; scale bar 100 µm. Note that there is an discret increase compare to control in the proportion of GFAP + cells distributed in the cell body of flat polygonal cells in cultures exposed to 0.1 µM FAB, with some polygonal cells co-expressing β-tub, typical of neural progenitor cells. Such effect was not observed at the extension in cultures exposed to 1 µM FAB. **(B)** Quantification of the proportion of GFAP + cells and β-tub + cells related to the total of cell nuclei counted by DAPI-stained nucleus (blue); the results are expressed as the percentage of means ± SD related to control, considered as 100%, in three independent experiments and were analyzed by Kruskal–Wallis ANOVA, followed by Turkey’s test for multiple comparisons (*) representing *p* ≤ 0.05 compared to control; **(C)** Morphological analysis of rMSCs maintained 21 days in control conditions (0.01% DMSO) or treated with a single dose of 1 µM FAB; obj. x20, scale bar 100 µm. In the inserts of image at obj. 40x, one can see some cells with neuronal morphology, presenting cellular process similar to neurites and interacting with other cells. **(D)** Photomicrographies of cultures in control conditions (0.01% DMSO) and 72 h after treatment with agathisflavone (1 µM FAB); immunocytochemistry, obj. x20, scale bar 100 µm. Note that there is an increase in the proportion of cells co-expressing GFAP and β-tub compared to control cultures, which is confirmed in **(E)** by quantifying the proportion of GFAP+/β-tub + cells, related to the total of cell nuclei; the results are expressed as the means of percentage in three independent experiments and analyzed by Kruskal–Wallis ANOVA followed by Turkey’s test for multiple comparisons (*) representing *p* ≤ 0.05 compared to control.

In order to determine whether the morphogenic effects of agathisflavone on rMSCs were associated with induction of differentiation, we performed immunocytochemistry for the astrocyte marker GFAP and the neuronal marker β-TubIII, 72 h after treatment with 0.1 or 1 µM agathisflavone or 0.01% DMSO vehicle in controls (Figure 3). In control cultures, the majority of rMSC were GFAP immunopositive, but labeling was restricted to the perinuclear region, whereas very few cells showed low β-TubIII expression (Figure 3A). In contrast, there was an evident increase in the intensity of GFAP and β-TubIII immunolabeling in rMSC cultures treated with 0.1 µM, but not observed with 1 µM agathisflavone treatment. The same analysis was performed in cultures 21 days after treatment with agathisflavone and it was determined that the majority of rMSC treated with 1 µM agathisflavone were immunopositive for GFAP and β-TubIII (Figures 3C–E). The results indicate that agathisflavone promotes neural differentiation of rMSC.

### Effects of Agathisflavone and rMSCs Treatment on Spinal Cord Injury (SCI)

#### Functional Motor Recovery

**FIGURE 4:**
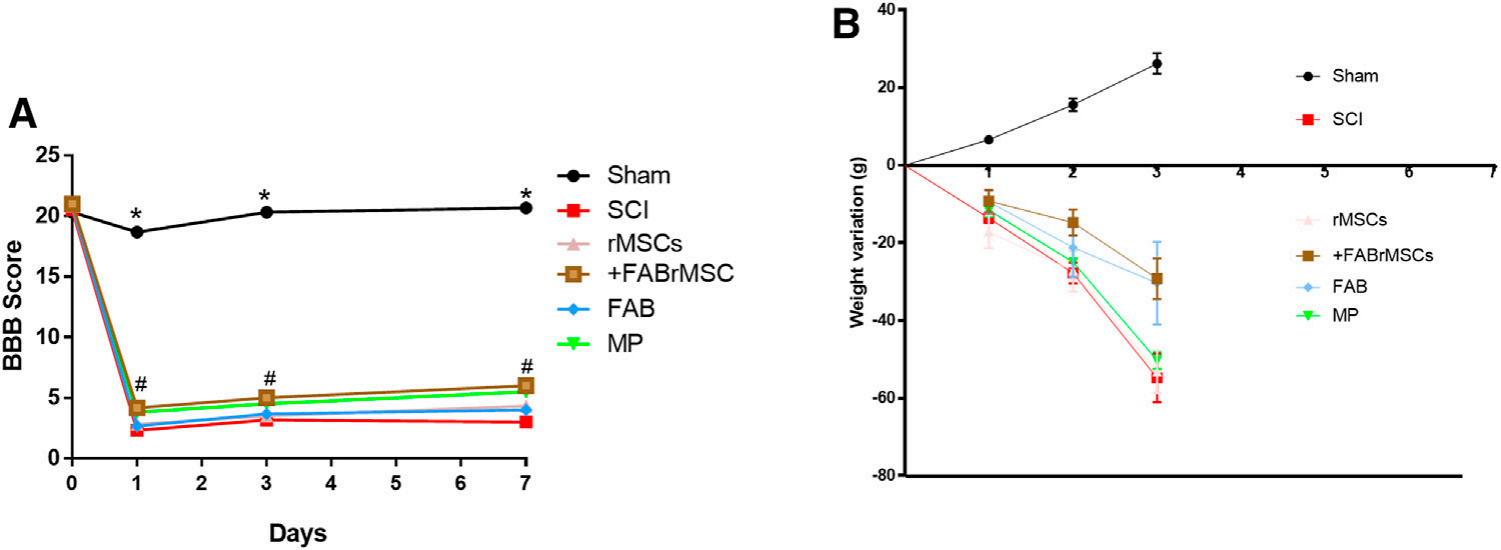
Behavioral outcomes in animals subjected to spinal cord injury (SCI). **(A)** Motor function assessment based on the Basso, Beattie, Bresnahan scale (BBB) on day zero (before SCI), day 1, day 3 and day 7 after SCI. Adult male Wistar rats (*n* = 6/group) underwent acute SCI and after 4 h were treated with a single application of 1 × 10^6^ control rMSCs or 1 × 10^6^ rMSCs pretreated with agathisflavone (+FABrMSCs), treated with one single dose of methylprednisolone (60 mg/kg i.p., MP), or treated daily with agathisflavone (10 mg/kg i. p., FAB) (#) *p* ≤ 0.05 vs. SCI rats. Data are the means ± SD **(B)** Weight variations of animals with SCI and different treatments.

Following the evaluation of the effects of agathisflavone on cultured rMSC, we examined the effects of its administration on the outcome of SCI, besides treatment of animals with the flavonoid. There were six experimental groups (*n* = 6 rats/group): 1) sham; 2) spinal cord injury (SCI); 3) SCI treated daily with agathisflavone (10 mg/kg i.p.); 4) SCI treated with control rMSCs; 5) SCI treated with agathisflavone treated rMSC (FABrMSCs); 6) SCI treated with methylprednisolone (MP, 60 mg/kg i.p.). Locomotor skills were assessed by the open-field test prior to SCI (day 0) and one, three and 7 days after SCI using the BBB score (Figure 4A; Table 2). Rats submitted only to laminectomy (Sham) recovered a score of 21 within 7 days after injury, which reflects no neurological impairments. Following SCI, rats achieved a mean BBB score of 3.0 ± 0.29 without treatment and that was not sigificantly altered by a single treatment with rMSC, which attained a mean BBB score of 3.0 ± 0.20 7 days after injury (DPI); both these experimental groups remained generally more apathetic than did sham controls. In contrast, the BBB score was significantly improved in the SCI + FABrMSC treatment group, attaining 5.36 ± 0.49 at 7 DPI; in addition, some of the animals had a trend towards improved motor function, compared to the other groups, with slight hind limb reflexes and rapid movement over the open field, as well as presenting grooming behavior. The motor behavior was very heterogeneous in the group of animals with SCI and treated with one single dose of methylprednisolone (60 mg/kg i.p.,MP). One animal presented being apathetic in the first 3 days after injury, the other animals, although injured, moved when stimulated in the open field reaching a BBB score of 4.8 ± 0.16. The animals with SCI and treated daily with agathisflavone (10 mg/kg i.p., FAB) presented similar behavior to the animals treated with MP, with the mean BBB score of 4.4 ± 0.32; they moved only when stimulated and an animal was shown to lean on its right side.

**TABLE 2:** Comparison between BBB scores. Data are expressed as means ± SD.

Day	Sham	SCI	rMSCs	rMSCs	+FABrMSCs	MP
1	20	2.7	2.8	4	4.8	4.6
3	20	2.9	3	4.5	5.0	4.8
7	21	3.4	3.3	4.8	5.3	5
Mean ± SD	20,33 ± 05,773	3 ± 1.3	3 ± 1.3	4.43 ± 1.0	5.03 ± 1.0	4.8 ± 0.8

SD, standard deviation.

Probability obtained from Student’s t-test.

Mean of the samples (α = 0.05).

#### Body Weight Change

After SCI, the animals were placed in cages with water and food *ad libitum*, and their weight gain or loss was monitored as a measure of general health, on the days on which the animals underwent motor evaluation (Figure 4B; Table 3). Control (Sham) animals gained approximately 30 g over the 7 days, with an average daily gain of 4.3 g. Animals with SCI presented an average daily loss of 8.53 g (Table 3). The groups of animals treated with rMSCs pretreated with agathisflavone (+FABrMSCs), or with MP or with FAB, presented an average daily loss of approximately 4.3 g. There are no statistical differences between these groups.

**TABLE 3:** Comparison of weight variation between SCI groups. Average daily weight loss/gain over 7 days.

Day	Sham	SCI	rMSCs	FAB	+FABrMSCs	MP
1	5.3 g	−8.51 g	−5.8 g	−5.5 g	−4.6 g	−4.3 g
3	12.9 g	−25.6 g	−13.l g	−13.7 g	−13.3 g	−12.8 g
7	29.8 g	59.71 g	−30.6 g	−30.l g	−29.8 g	−30.2 g
Mean	4.257 g	−8.53 g	−4.371 g	−4.3 g	−4.257 g	−4.314 g

#### Histological Changes

**FIGURE 5:**
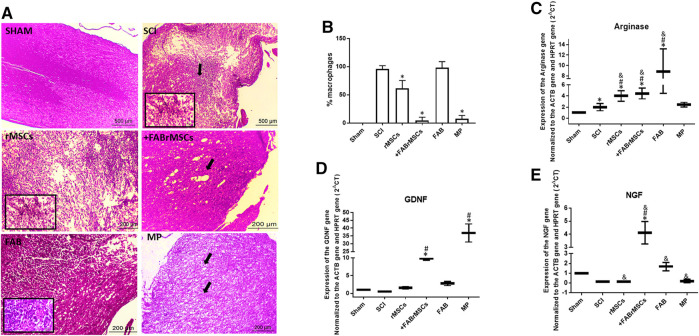
General histopathology of the spinal cord of animals 8 days after being subjected to spinal cord injury (SCI) and different treatments. **(A)** Representative longitudinal section of a normal spinal cord (sham animals), and from animals treated with a single application of 1 × 10^6^ control rMSCs or 1 × 10^6^ rMSCs pretreated with agathisflavone (+FABrMSCs), treated with one single dose of methylprednisolone (60 mg/kg i.p., MP), or treated daily with agathisflavone (10 mg/kg i.p., FAB); hematoxylin and eosin (H&E), x40; details x100. Abundant foamy macrophages and extensive area of liquefactive necrosis with strong macrophage reaction are observed in the spinal cord of animals with SCI (spotlight), also observed in the spinal cord of animals that received implant of control rMSC (spotlight), and in less expansion of animals treated daily with FAB. However, in the spinal cord of animals that received implant of +FABrMSCs, isolated vacuolization by demyelization and walerian degeneration (arrow) are observed. In the spinal cord of animals treated with MP, diffuse and mild vacuolization by demyelination of white matter is observed. **(B)** Quantification of foamy macrophages in injured spinal cord tissue. The results are expressed as the media of percentage in three independent experiments and were analyzed by Kruskal–Wallis ANOVA followed by Dunn’s post-test (*) *p* ≤ 0.05 compared to SCI group. **(C–E)**: Expression of neurotrophic factors and arginase in the spinal cord of animals 8 days after being subjected to spinal cord injury (SCI) and different treatments. Expression of mRNA for neurotrophic factors NGF and GFDN, and for enzyme arginase, was analyzed with RT-qPCR; Sham = Non-lesioned; SCI and other groups = Lesioned; values expressed as mean ± standard deviation; significant differences are expressed as *p ≤ 0.05 when compared to the control NL; #*p* ≤ 0.05 when compared to FAB 0.1 μM NL treatment; and *p* ≤ 0.05 when compared to the control L; and &*p* ≤ 0.05 when compared to FAB 0.1 μM treatment. Kruskal–Wallis and one-way ANOVA followed by Dunn’s post-hoc were used.

All animals were sacrificed 8 days after treatments for H&E histological analysis of the spinal cord (Figure 5A). The degree of lesion was determined according to the semi quantitative classification system, described by Ustun et al. (2014) (Table 4). Control (sham) animals did not present any tissue disruption of the spinal cord; both white matter and gray matter were intact, with no necrosis, inflammatory infiltrate or hemorrhage, classified with grade 0 of injury when comparing Ustun’s H&E histological analysis classification in Tables 4, 5. Animals with SCI presented an extensive area of liquefactive necrosis with the presence of severe hemorrhage, and numerous spongy macrophages at the lesion site, which was classified as Grade 3 of injury. Similar lesions were observed in the spinal cord of animals that received an implant of control rMSC, which had a mean score of 2.66 ± 0.47 (Table 5). In contrast, the group of animals treated daily with FAB presented injuries classified in Grade 2 and 3 depending on the tissue of animal analyzed. Animals treated with MP presented diffuse and mild vacuolization due to white matter demyelination in the spinal cord and the lesions were also classified as of Grade 2. However, animals that received a single application of +FABrMSCs presented spinal cords with walerian degeneration and isolated vacuolization by white matter demyelination vacuolations, and lesions classified in the majority as of Grade 2, demonstrating that agathisflavone-treated rMSC had potential to repair spinal cord injury. Moreover, quantification of foamy macrophages showed a significant reduction in the proportion of macrophages in the lesioned area in animals treated with rMSCs, and mainly in animals treated with +FABrMSCs and the anti-inflammatory MP.

**TABLE 4:** A semi-quantitative grading system according to Ustun et al. (2014). Spinal cord segment was examined for hemorrhage, spongiosis and liquefactive necrosis semi-quantatively for histopathological changes.

	Grade
No abnormal cells and change	0
Mild hemorrhage, spongiosis	1
Moderate hemorrhage and spongiosis with liquefactive necrosis	2
Some hemorrhage and spongiosis with glial cell proliferation and liquefactive necrosis	3

According Ustun et al. (2014).

**TABLE 5:** Histopathological evaluation by semi-quantitative Ustun classification system.

Rat	Sham	SCI	FAB	rMSCs	+FABrMSCs	MP
1	0	3	3	3	2	2
2	0	3	2	2	1	2
3	u	3	l	3	l	l
Mean ± SD	—	3 ± 0.0	2.33 ± 0.47	2.66 ± 0.47	1.66 ± 0.47	2.0 ± 0.0
*p*	—	—	0.198	0.0153	0.0377	—

SD: standard deviation.

Mean of the samples. * significance level α = 0.05.

#### Gene Expression Changes

In order to better characterize the inflammatory profile in the site of lesion, RT-qPCR was performed with samples of the site of SCI to analyze expression of neurotrophins and arginase, an enzyme directly related to the M2 anti-inflammatory profile of macrophages (Figures 5B–E). A significant increase in nerve growth factor (NGF) mRNA expression was observed in the spinal cord tissue of animals with SCI treated with +FABrMSCs (about 10 times) or FAB (about 2 times) compared with animals with the lesion. Moreover, a significant increase in mRNA expression for glial-derived growth factor (GDNF) was also observed in the spinal cord tissue of animals with SCI treated with rMSCs (about 2 times), +FABrMSCs (about 10 times) or FAB (about 3 times) compared with animals with the lesion. Aghathisflavone (FAB) treatment also induced a significant increase (about 8 times) in mRNA expression for the enzyme arginase, a tendency also observed in the spinal cord tissue of animals with SCI treated with rMSCs (about 3.7 times), +FABrMSCs (about 4.2 times). Treatment of animals with MP induced a decrease in NGF and an increase in GDNF compared with the tissue of control animals without lesion (Sham).

## Discussion

Research with MSCs has shown that these cells can differentiate in specific conditions into neural lineages and could be used in clinical situations such as SCI (Foudah et al., 2014; Assinck et al., 2017; Jin et al., 2019). In addition, the phytoestrogen agathisflavone has prominent neuroprotective effects and is capable of inducing neurogenesis in embryonic and pluripotent stem cells (Paulsen et al., 2011; Andrade et al., 2018). Studies in the literature show the neuroprotective activity of flavonoids, including the ability of these compounds to cross the blood-brain barrier. Rutin and quercetin (Ferri et al., 2015), hesperetin and naringenin (Youdim et al., 2003), and polyphenols in general (Figueira et al., 2017), which suggests the ability of agathisflavone to also act directly enter the cerebrospinal fluid for direct efficacy alone or combinated with other molecules. Here, we show that the treatment of rMSCs cells with agathisflavone promotes a neural phenotype differentiation *in vitro* in a small population, reinforcing the heterogeneity of MSC subpopulations.

According to Neuhuber et al. (2008), rMSCs naturally present heterogeneous morphology. However, this heterogeneity is related to the “stemness” of these cells. According to these authors, rMSCs cells that have a fusiform or fibroblastic morphology have multipotent characteristics and, therefore, this morphology is characterized as immature (Sekiya et al., 2002). Large cells with a flat polygonal morphology and a clearly visible nucleus without processes, or with very short processes, were classified as “smaller stem potential” or mature cells (Colter et al., 2001; Prockop et al., 2001; Sekiya et al., 2002; Neuhuber et al., 2008).

Depending on the agathisflavone concentration and DIV, rMSCs retained the two characteristic morphological profiles typical of immature multipotency. Treating with the flavonoid at the concentration of 1 µM, the proportion of cells with long and thin process was increased, and that may be indicative of flavonoid modulation in these cells to a more mature neuronal profile, as we described previously in embryonic stem cell cultures (Paulsen et al., 2011).

Spinal cord injury is characterized by primary events and secondary events. Primary events refer to loss of spinal cord integrity due to mechanical factors. Late secondary damage refers to a complex set of pathophysiological processes including ischemia, edema, inflammation, excitotoxicity, oxidative cell damage and digestive system complications with changes in nutrient absorption and intestinal motility (Zhang and Liao*,* 2014; Hakim et al., 2019). Consequently, there is glial scar formation and neurological dysfunction (Byrnes et al., 2007; Bradbury and Burnside, 2019).

Secondary injury begins within minutes of the initial primary injury and continues for weeks or months causing progressive damage to the spinal cord around the injury site. The secondary lesion may be temporarily divided into acute, subacute and chronic phases. The acute phase begins immediately after SC and includes vascular damage, ionic imbalance, neurotransmitter accumulation (excitotoxicity), free radical formation, inflammation, edema and necrotic cell death (Oyinbo, 2011; von Leden et al., 2016; Alizadeh et al., 2019). Demyelination of white matter, liquefactory necrosis and the presence of spongy macrophages also reflect secondary SCI (Oyinbo, 2011).

Corticosteroids, especially methylprednisolone (MP), have the potential to stabilize cell membrane structure by maintaining an intact blood-brain barrier, reducing vasogenic edema, decreasing medullary blood flow, altering electrolyte concentrations at the site of injury, inhibiting endorphin release, decreasing free radical damage and limiting post-traumatic inflammatory response (Wang et al., 2019). However, their use has little success on motor and functional recovery and numerous efforts have been made by the scientific community regarding cell therapy for motor and functional recovery and the use of new drugs to reduce inflammation and tissue destruction in the spinal cord injury environment as an alternative to MP. Hence, as an alternative, the implant of MSCs has been largely used, both *in situ* and administered via the tail vein in view to evaluate tissue recovery and inflammatory response at the injury site (Leypold et al., 2007; Hakim et al., 2019). Moreover, there are growing evidences that the product of secretion of MSCs is the major responsible for restoring tissue in models of SCI. In this context, in the present study, we adopted two strategies in male Wistar rats subject to acute SCI: a single application (via caudal vein) of control rMSCs and with a differentiated profile induced by the agathisflavone treatment *in vitro* before implant. Treatment of animals with 21-day agathisflavone-treated rMSCs was able to protect the injured spinal cord tissue and improve motor functions (with the highest BBB score), effects that are associated with the increase in expression of NGF, GDNF and arginase, and reduction on the macrophage infiltrate. Treatment of animals with agathisflavone alone (10 mg/kg) was also able to protect injured spinal cord tissue, increase the expression of neurotrophins and modulate the inflammatory response.

Zhang et al. (2014) demonstrated that the agathisflavone monomer apigenin (10 mg/kg) in a SCI model improved neurological recovery after injury, obtaining results like treatment with MP, neuroprotective effect that was at least partially associated with its antioxidant and anti-inflammatory effects. In the present study, treatment with MP presented results like those described by Zhang et al. (2014) and by Leypold et al. (2007), who demonstrate that MP, when administered in the initial period of spinal cord injury, may decrease the extent of spinal cord hemorrhage, reducing secondary damage caused by injury protecting motor functions. In a study developed by Shi et al. (2016), the flavonoid narigenin, repeatedly administered at higher concentrations (100 mg/kg), protected animals from SCI damages. That happened possibly due to the inhibition of inflammation via miR 233, a known microRna associated with inflammatory response that is a product of secretion that could be investigated, besides othe miRNAs, in rMSCs treated with agathisflavone and in the area of the damage. The ensemble of the results showed that rMSC had the potential to repair the injured cord. It is possible, however, that due to the fact that the observation window of the study is short, only 7 days, it was not possible to verify this tissue recovery and motor improvement in animals treated with rMSCs.

Nevertheless, the group of animas that receive agathisflavone pre-treated rMSCs presented lower tissue injury and motor improvement, when compared to the other groups, effects also associated with the increase in expression of NGF and GDNF, besides the attenuation of the inflammatory infiltrate. This increase in neurotrophins expression is very interesting, since NGF has demonstrated neuroprotective role in the recovery of SCIs, and is related to the inhibition of stress-induced cell death of reactive oxygen species by activating signals in the regulation of inflammatory process (Zhang and Liao, 2014); also, Keefe et al. (2017) showed that NGF expression in the spinal cord induced the growth of nociceptive axons and hyperalgesia in animals with SCI. On the other hand, GDNF is also known to be an important neurotrophic factor for CNS development because it promotes neuronal survival and axonal regeneration, reduces secondary damage, decreases lesion size and improves functional recovery (Ortmann and Hellenbrand, 2018); GDNF, when expressed in traumatic injuries, favors nerve fiber growth and improves motor effects of injured animals (Pöyhönen et al., 2019). Together, these findings corroborate the results of the present study, which showed improvement in motor function and preservation of the spinal cord tissue associated with GDNF and NGF.

Finally, another important finding in the present study was the increase in the expression of the enzyme arginase in the injured tissue of animals that received treatment with the flavonoid agathisflavona alone or flavonoid treated-MSCs; also, the last case was associated with a drastic reduction on the macrophage infiltrate in the injured tissue, compared with animals without treatments. Arginase expression is related to the activation of M2 antiiflamatory profile of macrophages. Macrophages could promote repair of injured tissue by regulating transitions through different phases of the healing response and facilitating transitions through inflammatory, proliferative and repair remodeling phases (Ahn et al., 2012; Gensel and Zhang, 2015). In this sense, treatment with agathisflavone-treated rMSCs presented advantages when compared to the other groups, because it protects the injured spinal cord tissue, evidenced by the histopathological findings, as well as by the modulation expression of neurotrophins and neuroinflammation, which appears to be related to the modulation of releasing factors in the flavonoid-treated mesenchymal stem.

## Conclusion

All these findings together demonstrated that the flavonoid agathisflavone was not toxic to rMSCs, and induced these cells to a neural differentiated profile, with increased expression of neural markers GFAP and β-tubulin III. Moreover, in the SCI model, agathisflavone alone at the doses tested was unable to protect completely the injured spinal cord tissue, but increased the expression of neurotrophins that are related to nerve growth and increased arginase expression suggesting activation of the anti-inflammatory M2 macrophage profile. In addition, the administration of agathisflavone-treated rMSCs showed anti-inflammatory properties, protecting injured spinal cord tissue, also increasing NGF and GDNF expression, reflecting improved motor function, ensemble of results that may be considered in further studies for clinical application.

## Data Availability

The original contributions presented in the study are included in the article/Supplementary Material, further inquiries can be directed to the corresponding author.

## Supplementary Material

Supplementary Figure S1 Characterization of rat mesenchymal stem cells (rMSCs). **(A)** Morphological aspect of 15 days *in vitro* (DIV). **(B)** rMSC differentiation characterization in adipocytes by Oil red O staining. **(C)** differentiation characterization in osteocytes by alizarine red staining.

Supplementary Figure S2
**Supplementary Figure S2** | Characterization of rat mesenchymal stem cells (rMSCs) by flow cytometry surface markers for the *Ratus novergicus.*

## Glossary

BBB: Basso, Beattie, Bresnahan scale
BSA: bovine serum albumin
CD11b: integrin alpha M
CD29: integrin beta 1
CD34: transmembrane phosphoglycoprotein protein cluster
CD73: ecto-5′-nucleotidase
CD90: VLA-β integrin
cDNA: complementary Deoxyribonucleic acid
DMEM: Dulbecco’s Modified Eagle Medium
DMSO: dimethyl sulfoxide
FAB: agathisflavone
FBS: fetal bovine serum
GDNF: glia-derived neurotrophic factor
GFAP: glial fibrillary acidic protein
H&E: hematoxylin and eosin
HPRT1: hypoxanthine phosphoribosyltransferase 1
IP: intra peritoneal
MHC-II: histocompatibility complex class II
miR: microRNA
MP: methylprednisolone
MSCs: mesenchymal stem cells
MTT assay: 3-(4,5-dimethylthiazol-2-yl)-2,5-diphenyltetrazolium bromide test
NGF: nerve growth factor
PBS: Phosphate-buffered saline
PFA: paraformaldehyde
rMSC: rat mesenchymal stem cells
RNA: ribonucleic acid
RT: room temperature
RT-qPCR: quantitative real-time polymerase chain reaction
SCI: spinal cord injury
Triton X-100: 4- (1,1,3,3-tetramethylbutyl) phenyl polyethylene glycol
