# Supplementary figures and images for: Agathisflavone as a Single Therapy or in Association With Mesenchymal Stem Cells Improves Tissue Repair in a Spinal Cord Injury Model in Rats
Source: Front Pharmacol. 2022 Apr 5;13:858190. doi: 10.3389/fphar.2022.858190 (PMC9037239; doi:10.3389/fphar.2022.858190)

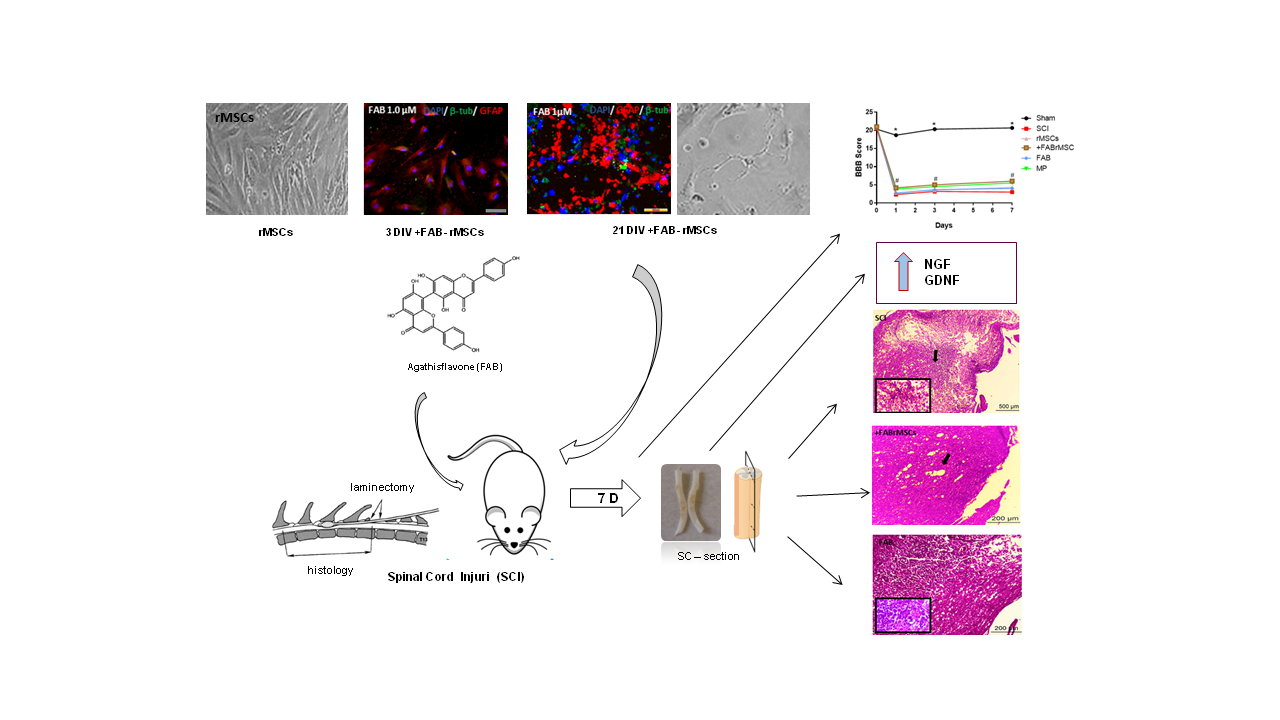

Supplement: Supplementary file 1 [file Image3.TIF]

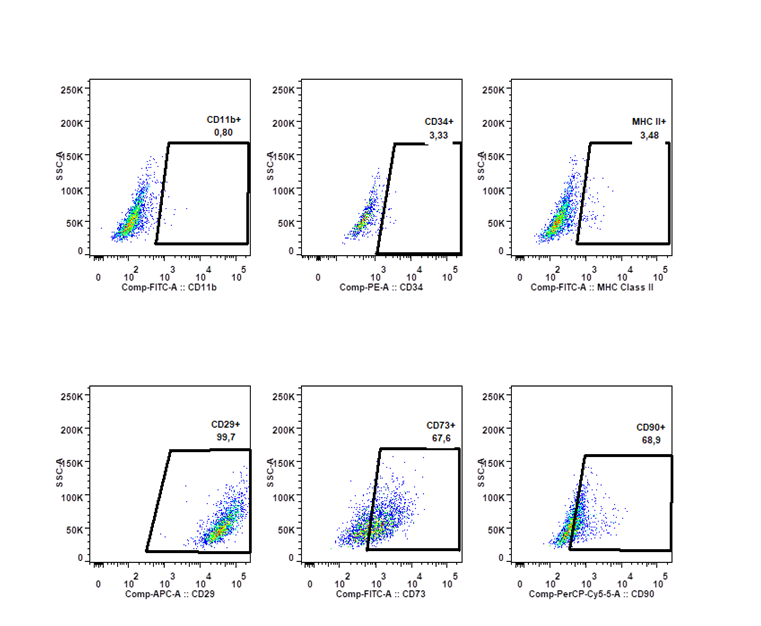

Supplement: Supplementary file 2 [file Image2.TIF]

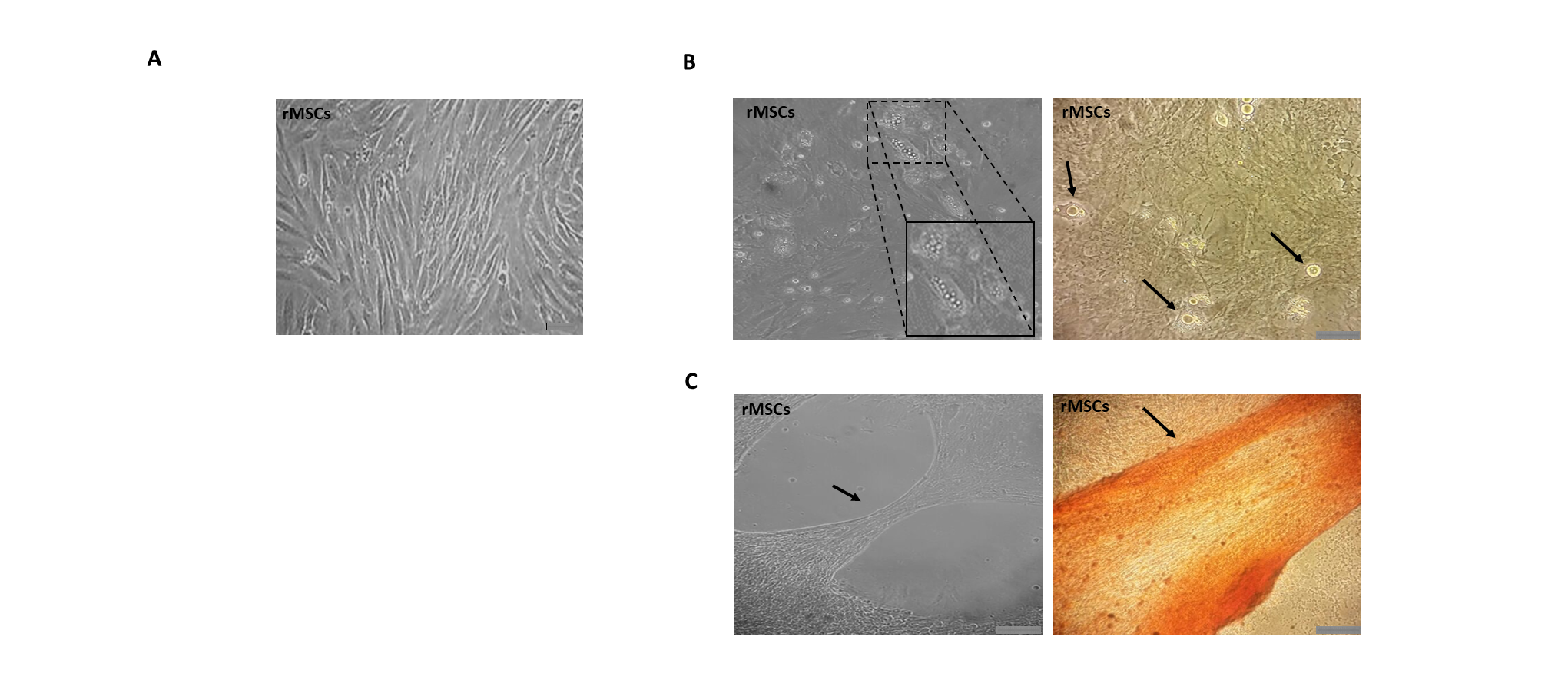

Supplement: Supplementary file 3 [file Image1.TIF]
